# A portable mnemonic to facilitate checking for cognitive errors

**DOI:** 10.1186/s13104-016-2249-2

**Published:** 2016-09-17

**Authors:** Keng Sheng Chew, Jeroen van Merriënboer, Steven J. Durning

**Affiliations:** 1 Faculty of Medicine and Health Sciences, Universiti Malaysia Sarawak, Kota Samarahan, Malaysia; 2 School of Health Education, Maastricht University, Maastricht, The Netherlands; 3 Uniformed Services University of the Health Sciences, Bethesda, USA

## Abstract

**Background:**

Although a clinician may have the intention of carrying out strategies to reduce cognitive errors, this intention may not be realized especially under heavy workload situations or following a period of interruptions. Implementing strategies to reduce cognitive errors in clinical setting may be facilitated by a portable mnemonic in the form of a checklist.

**Methods:**

A 2-stage approach using both qualitative and quantitative methods was used in the development and evaluation of a mnemonic checklist. In the development stage, a focus-driven literature search and a face-to-face discussion with a content expert in cognitive errors were carried out. Categories of cognitive errors addressed and represented in the checklist were identified. In the judgment stage, the face and content validity of the categories of cognitive errors represented in the checklist were determined. This was accomplished through coding responses of a panel of experts in cognitive errors.

**Results:**

From the development stage, a preliminary version of the checklist in the form of four questions represented by four specific letters was developed. The letter ‘T’ in the TWED checklist stands for ‘Threat’ (i.e., ‘is there any life or limb threat that I need to rule out in this patient?’), ‘W’ for ‘Wrong/What else’ (i.e., ‘What if I am wrong? What else could it be?’), ‘E’ for ‘evidences’ (i.e., ‘Do I have sufficient evidences to support or exclude this diagnosis?’), and ‘D’ for ‘dispositional factors’ (i.e., ‘is there any dispositional factor that influence my decision’). In the judgment stage, the content validity of most categories of cognitive errors addressed in the checklist was rated highly in terms of their relevance and representativeness (with modified kappa values ranging from 0.65 to 1.0). Based on the coding of responses from seven experts, this checklist was shown to be sufficiently comprehensive to activate the implementation intention of checking cognitive errors.

**Conclusion:**

The TWED checklist is a portable mnemonic checklist that can be used to activate implementation intentions for checking cognitive errors in clinical settings. While its mnemonic structure eases recall, its brevity makes it portable for quick application in every clinical case until it becomes habitual in daily clinical practice.

**Electronic supplementary material:**

The online version of this article (doi:10.1186/s13104-016-2249-2) contains supplementary material, which is available to authorized users.

## Background

Striving to make an accurate diagnosis using sound clinical decision making skills is undoubtedly the goal of every clinician. In reality though, diagnostic error rates range from 5 to 15 % [1, 2]. Although the root causes of diagnostic errors are often multi-factorial, a large proportion of these errors have cognitive components [3, 4]. With sufficient training and experience, a clinician acquires a large repertoire of illness representation models known as ‘illness scripts’ [5]. Illness scripts allow a clinician to make fast and accurate clinical decisions via pattern recognition [5–7]. However, while using pattern recognition results in accurate diagnoses most of the time [2, 8], there are occasions when such pattern recognition may derail the clinician into cognitive errors [9, 10] such as anchoring bias [11]. Anchoring bias occurs when the illness “pattern” recognized at the outset of the diagnostic process results in the clinician’s fixation on this initial impression so much so that the clinician fails to adjust this initial impression even in the light of contradicting additional data [11]. Numerous strategies have been suggested for overcoming these cognitive errors [11, 12] but a key question remains whether the clinician implements these strategies, particularly in a busy clinical setting.

According to Gollwitzer [13, 14], people who are absorbed in their on-going tasks may find it difficult to implement their intended goals. In particular, clinicians who are absorbed in highly demanding clinical tasks, such as managing emergency cases or having to attend to multiple patients at the same time may simply not remember to carry out the intention of minimizing cognitive errors. In other words, merely having the intention is not sufficient to ensure its implementation. One of the reasons for this intention-implementation gap is because clinician forgets to act on the intended task [14]. The ability to remember acting on a postponed intended task following a period of interruptions is known as prospective memory [15, 16]. Unfortunately, during a clinical emergency, time and cognitive resources are limited. When under stress, the clinician’s memory becomes increasingly unreliable leading to prospective memory failure especially if the intended task is not part of one’s routine activities [14, 17–19]. Nonetheless, this intended task of reducing cognitive errors can become more attainable when a person explicitly incorporates specific implementation intentions in their clinical work [13]. Implementation intentions are the cognitive “if–then” steps that serve to bridge the intention-implementation gap [13, 14].

Using a checklist can timely activate this “if–then” step of implementation intention [15]. For example, the clinician may say, “*If* I have arrived at an initial diagnostic impression, *then* I must remember to ask myself these questions aimed to reduce cognitive errors”. The linchpin of mnemonic checklist is that it eases recall and overcomes the barrier of prospective memory failure, particularly in a stressful clinical environment [15, 16].

This paper describes the two-stage approach in the development and evaluation of such a mnemonic checklist with the objective of aiding prospective memory in checking cognitive errors. Both qualitative and quantitative research methods were incorporated. Institutional research and ethics committee approval was obtained prior to starting this study.

## Methods

### Participants

To quantitatively assess the content validity of the TWED checklist, ten senior emergency physicians with more than 5 years’ experience were invited as judges. Eight of them responded. These judges were first given the instructions for the evaluation task. This task included the use of an assessment form to evaluate the representativeness of these categories of cognitive errors in the respective quadrants of the TWED checklist, as well as their relevance in a clinical setting by using four-point Likert scales. The judges performed their evaluations independently. They were between 35 and 45 years old (mean age = 38.5, *SD* = 3.16 years), 5 out of 8 (62.5 %) of them were male, years of clinical experience varied between 9 and 19 years (mean years = 12, *SD* = 3.16 years).

Whereas, to verify the face validity and applicability of the checklist, seven experts on cognitive errors in clinical decision making were invited. They were invited via emails and all seven of them agreed to participate. Their expertise and clinical positions are listed in the Additional file 1.

### Procedure

In the development stage, a focus-driven search was conducted via Web of Science and Google Scholar using the keywords of “diagnostic error” OR “cognitive error” OR “cognitive bias” AND “checklist” to specifically answer the following questions: Is there any form of classification or category of common cognitive errors in clinical setting that has been developed? (These categories of cognitive errors would then be used as the basis to develop this mnemonic checklist). Are there any comparative checklists aimed to reduce diagnostic errors that have already been developed and published? Are these checklists formatted in mnemonic format? A mnemonic format checklist is one where specific letters or keywords are framed to represent the items in the checklist in order to ease prospective memory while a non-mnemonic checklist is one where the items are listed without incorporation of any memory aid that can activate implementation intentions.

Search was limited to English language articles only from year 2008 onwards. Only articles that describe some form of classification or categories of cognitive errors and articles that describe checklists aimed to reduce cognitive errors in a clinical setting were selected. Articles that merely describe individual cognitive errors without any form of classification were excluded. Articles addressing cognitive errors in non-clinical settings were also excluded. After the categories of common cognitive errors in diagnostic errors were identified, the checklist was then drafted and several sessions of face-to-face discussions and conversation with a content expert (PC) in cognitive errors was carried out to verify the importance of these categories in clinical setting. This content expert was chosen based on his contributions in this area of cognitive errors in clinical decision making including some of the articles referenced here [1, 8, 10, 11]. One of the authors [KSC] first contacted this content expert and spent over a period of 3 months with him on how he conducted education and trainings in this area of cognitive errors in clinical decision making and efforts to minimize them.

In the judgment stage, the content validation and the applicability of the checklist were determined (Table 1). To assess the content validity of the categories of cognitive errors represented in the checklist, content validity index (CVI) and the modified kappa were used. In particular, the representativeness and relevance of these categories of cognitive errors were determined. The CVI for relevance and representativeness is defined as the proportion of the judges who rate the item with scores of 3 or 4 on a four-point Likert scale (for representativeness: 1 = not representative of the quadrant, 2 = somewhat representative, 3 = quite representative, 4 = highly representative; and for relevance: 1 = not relevant at all, 2 = somewhat relevant, 3 = quite relevant, and 4 = highly relevant) [17]. The content validity for the entire checklist was then calculated by averaging the CVIs of each individual category [17]. Each of these categories was marked as an item in the CVI analysis. To account for chance agreement, modified kappa statistics [18] for each item were calculated as well.

**Table 1:** List of generic checklists aimed to minimize cognitive errors in clinical setting

Author(s), Year	Description	Reference
Graber et al. (2014)	A generic checklist developed to reduce risk of diagnostic errors. The checklist consists of 2 parts. The first part contains 10 reflective questions aimed to identify high risk situations for diagnostic error: (1) Are there “must-not-miss” diagnoses that need consideration? (2) Did I just accept the first diagnosis that came to mind? (3) Was the diagnosis suggested to me by the patient, nurse, or another MD? (4) Is there data about this patient I haven’t obtained and reviewed? Old records? Family? Primary care provider? (5) Are there any piece pieces that don’t fit? (6) Did I read the X-ray myself? (7) Was this patient handed off to me from a previous shift? (8) Was this patient seen in the ER or clinic recently for the same problem? (9) Was I interrupted/distracted/cognitively overloaded while evaluating this patient? (10) Is this a patient I don’t like for some reason? Or like too much? (friend, relative). A “Yes” response to any of the questions puts the clinician at high risk for error. The second part suggests 3 things to do in high risk situations: (1) Pause to reflect—take a diagnostic “time-out” (2) Consider the universal antidote: What else could this be? (3) Make sure the patient knows when and how to get back to you if necessary	[20]
Ely et al. (2011)	This generic checklist consists of 5 steps: (1) Obtain your own complete medical history (2) Perform a focused and purposeful physical exam (3) Generate and differentiate initial hypotheses with further history, physical exam, and diagnostic tests (4) Pause to reflect—take a diagnostic “time-out” by asking the following five questions: (a) Was I comprehensive? (b) Did I consider the inherent flaws of heuristic thinking (c) Was my judgment affected by any other bias? (d) Do I need to make the diagnosis now, or can I wait? (e) What is the worst-case scenario? (5) Embark on a plan, but acknowledge uncertainty and ensure a pathway for follow-up	[21]
Leo Leonidas’ ten commandments to reduce cognitive errors as quoted in Graber (2009)	The ten commandments are: (1) Thou shalt reflect on how you think and decide. (2) Thou shalt not rely on your memory when making critical decisions. (3) Thou shalt make your working environment information-friendly by using the latest wireless technology such as the tablet PC and PDA. (4) Thou shalt consider other possibilities even though you are sure of your first diagnosis. (5) Thou shalt know Bayesian probability and the epidemiology of the diseases in your differential diagnosis. (6) Thou shalt mentally rehearse common and serious conditions that you expect to see in your specialty. (7) Thou shalt ask yourself if you are the right person to make the final decision or a specialist after considering the patient’s values and wishes. (8) Thou shalt take time to decide and not be pressured by anyone. (9) Thou shalt create accountability procedures and follow up for decisions made. (10) Thou shalt record in a relational data base software your patient’s problems and decisions for review and improvement	[4]
Mamede et al. (2008)	A 11-step checklist aimed to improve diagnostic accuracy by reflective practice. The 11 steps are: (1) Read the case again (2) Write down the hypothesis previously indicated again (3) List findings that support this hypothesis (4) List findings that oppose it (5) List findings that would be expected if the hypothesis-at-hand were true but which were not encountered in the case. (6) List alternative hypotheses if the first hypothesis proved to be incorrect. For each of these alternative hypotheses generated from step (6), then (7) list findings consistent with the hypothesis (8) list findings that contradicted the hypothesis (9) list findings that were expected but not present in the case. Based on this analysis, then (10) indicate your conclusions by ranking diagnostic hypotheses in order of likelihood and (11) present a final diagnosis	[19]

To assess the face validity of the checklist as well as its applicability in clinical settings, a structured expert consultation via email communications was carried out. Responses from the experts were then coded by one of the authors (CH) using the NVivo for Mac software. Open coding was first performed in the software by repeated analytical readings and labeling keywords and phrases from these email responses, based on the five questions asked:

1. Are the important facets of preventing cognitive errors adequately covered in this mnemonic checklist?
2. When should this mnemonic checklist be used? Before formulating the initial diagnosis? Or after?
3. How often should this mnemonic checklist be used? For every case seen? Or only for selective cases? What types of cases, if selective?
4. Cognitive processes involved in medical decision making are highly complex. Does the use of a mnemonic checklist lead to oversimplification of the cognitive processes involved?
5. Is a mnemonic checklist mainly useful for novice clinicians, for more experienced ones, or for both groups?

After the open coding, axial coding was performed by re-analyzing these open codes to look for similarities, differences and relationships among these responses. The analyses were then sent back to the experts for member-checking before the final version of their detailed opinions was tabulated (see Additional file 1). Appropriate modifications based on the participants’ suggestions and comments were made accordingly.

## Results

From the focus-driven search, six articles were selected [4, 9, 19–22]. Six categories of common cognitive errors contributing to diagnostic errors were identified [9, 22]. Although not exhaustive, this classification addresses the important ones in clinical setting. They are: (1) errors due to over-attachment to a particular diagnosis (examples of cognitive biases in this class include anchoring and confirmation bias); (2) errors due to failure to consider alternative diagnoses (one example is search satisficing); (3) errors due to inheriting someone else’s thinking (for example, diagnostic momentum and framing effect); (4) errors in prevalence perception or estimation (for example, availability bias, gambler’s fallacy and posterior probability error); (5) errors involving patient characteristics or presentation context (for example, fundamental attribution error, gender bias), and (6) errors that are associated with the doctor’s affect or personality (for example, visceral bias and sunk cost fallacy). Previously published checklists aimed to reduce diagnostic errors have all been formatted in non-mnemonic format [4, 19–21].

A preliminary version of the checklist was developed after discussion with the content expert (PC). This checklist is divided into four quadrants with each quadrant posing an activation question represented by a letter. The letter ‘T’ stands for ‘Threat’ (i.e., ‘is there any life or limb threat that I need to rule out in this patient?’), ‘W’ for ‘Wrong/What else’ (i.e., ‘What if I am wrong? What else could it be?’), ‘E’ for ‘Evidences’ (i.e., ‘Do I have sufficient evidences to support or exclude this diagnosis?’), and ‘D’ for ‘Dispositional factors’ (i.e., ‘is there any dispositional factor that influences my decision’; and this quadrant is further divided into two groups of factors, represented by 2 ‘E’s: the ‘Environmental’ factors and the ‘Emotional’ factors’ which can come from the doctor or the patient. Hence the checklist was named the ‘TWED checklist’. The TWED checklist and the corresponding groups of cognitive errors addressed by each quadrant are given in Table 2.

**Table 2:** TWED checklist and the cognitive errors addressed

TWED Checklist	Classification of cognitive errors covered (based on Campbell et al. 2007)	Rationale
**T** = life or limb Threat (What are the life or limb threatening conditions in this patient?)	Cognitive errors due to failure to consider alternative diagnoses (worst-case scenarios)	This domain encapsulates the rule-out-worse-case scenarios (ROWS) heuristics as a form of cognitive forcing strategy
**W** = Wrong? (What if I am wrong? What else could it be?)	Cognitive errors due to overattachment to a particular diagnosis and cognitive errors due to failure to consider alternative diagnoses	This domain is to reduce the risk of committing cognitive errors such as search satisficing, anchoring, confirmation, availability biases, etc.
**E** = evidences (Do I have sufficient evidences for or against this diagnose?)	Cognitive errors due to inheriting someone else’s thinking and cognitive errors due to erroneous estimation or perception of prevalence	This domain is to minimize cognitive errors such as anchoring, confirmation bias, blind spot, myside bias, ego bias, etc.
**D** = dispositional factors (What are the environmental & emotional (2es) dispositions influencing my decision?)	Environment influences are not explicitly listed as one of the categories but discussed in this paper as error-producing conditions (EPC). These are high-pressured conditions that often exist by necessity, but are prone to cognitive errors because of the expectation to shorten the decision making process	These are the factors that may increase the risk of committing cognitive errors. These can be further divided into 2 ‘E’s: the environmental factors—e.g., chaotic, busy working place; and the ‘emotional factors’. These emotional factors can come from the physician or from the patient

In the judgment stage, the results for the content validity are given in Tables 3 and 4. Generally, most of these categories of cognitive errors were rated highly in terms of their relevance and representativeness (with a modified kappa value of 0.65–1.0), except for the relevance of two categories, namely, ‘cognitive errors due to erroneous estimation or perception of prevalence’ under the quadrant of “E = Evidences” (with a modified kappa value of 0.41) and ‘cognitive errors associated with patient characteristics (‘emotive’ influence of patient)’ under the quadrant of “D = Dispositional factors”. These two categories were rated as “fair” and “good” respectively although they were rated as “excellent” in terms of how well they are represented in their respective quadrants.

**Table 3:** CVI-Relevance and the modified kappa statistics (κ*) of item relevance

Quadrant and item	CVI-relevance	p_c_	Modified κ*	Evaluation of κ*
Quadrant 1: T = life or limb threat (What are the life or limb threatening conditions in this patient?)				
Item 1: cognitive errors due to failure to consider life or limb threatening conditions	1.0	0.008	1.0	Excellent
Quadrant 2: W = wrong? (What if i am wrong? what else could it be?)				
Item 2: cognitive errors due to overattachment to a particular diagnosis	1.0	0.008	1.0	Excellent
Item 3: cognitive errors due to failure to consider alternative diagnoses	1.0	0.008	1.0	Excellent
Quadrant 3: E = evidences (Do i have sufficient evidences for or against this diagnose?)				
Item 4: cognitive errors due to inheriting someone else’s thinking	0.86	0.055	0.85	Excellent
Item 5: cognitive errors due to erroneous estimation or perception of prevalence	0.57	0.273	0.41	Fair
Quadrant 4: D = dispositional factors (What are the environmental & emotional (2Es) dispositions influencing my decision?)				
Item 6: Cognitive errors associated with patient characteristics (‘emotive’ influence of patient)	0.71	0.164	0.65	Good
Item 7: Cognitive errors associated with doctor’s affect or personality (‘emotive’ influence of doctor)	0.86	0.055	0.86	Excellent
Item 8: Cognitive errors caused by impact of the workplace environment (‘environmental’)	0.86	0.055	0.86	Excellent

The formula for modified kappa statistic (κ*) = (CVI-Relevance − pc)/(1 − pc), where pc represents probability of a chance occurrence [18]

p_c_ is the probability of chance of occurrence. The formula for p_c_ is: N!/[A!*(N−A)!]*0.5 ^N^ where N = the number of judges, A = the number agreeing on good relevance [18]

Evaluation criteria for modified kappa (κ*): κ* = fair (0.40–0.59), κ* = good (0.60–0.74) and κ* = excellent (>0.74)

CVI should be 0.88 and above to establish validity with a p < 0.05

**Table 4:** CVI-representativeness and the modified kappa statistics (κ*) of item representativeness

Quadrant and item	CVI-representativeness	p_c_	Modified κ*	Evaluation of κ*
Quadrant 1: T = life or limb threat (What are the life or limb threatening conditions in this patient?)				
Item 1: cognitive errors due to failure to consider life or limb threatening conditions	1.0	0.008	1.0	Excellent
Quadrant 2: W = Wrong? (What if i am wrong? what else could it be?)				
Item 2: cognitive errors due to over attachment to a particular diagnosis	1.0	0.008	1.0	Excellent
Item 3: cognitive errors due to failure to consider alternative diagnoses	0.86	0.055	0.85	Excellent
Quadrant 3: E = evidences (Do I have sufficient evidences for or against this diagnose?)				
Item 4: cognitive errors due to inheriting someone else’s thinking	0.86	0.055	0.85	Excellent
Item 5: cognitive errors due to erroneous estimation or perception of prevalence	1.0	0.008	1.0	Excellent
Quadrant 4: D = dispositional factors (What are the environmental & emotional (2Es) dispositions influencing my decision?)				
Item 6: cognitive errors associated with patient characteristics (‘emotive’ influence of patient)	0.86	0.055	0.85	Excellent
Item 7: cognitive errors associated with doctor’s affect or personality (‘emotive’ influence of doctor)	1.0	0008	1.0	Excellent
Item 8: cognitive errors caused by impact of the workplace environment (‘environmental’)	1.0	0.008	1.0	Excellent

The formula for modified kappa statistic (κ*) = (CVI-Relevance − pc)/(1 − pc), where pc represents probability of a chance occurrence [18]

p_c_ is the probability of chance of occurrence. The formula for p_c_ is: N!/[A!*(N-A)!]*0.5 ^N^ where N = the number of judges, A = the number agreeing on good relevance [18]

Evaluation criteria for modified kappa (κ*): κ* = fair (0.40–0.59), κ* = good (0.60–0.74) and κ* = excellent (>0.74)

CVI should be 0.88 and above to establish validity with a p < 0.05

Based on the codings, five main findings regarding the face validity and applicability of TWED checklist were identified. Saturation reached after the analysis of 5–6 of these transcripts. First, the TWED checklist comprehensively although not exhaustively covers all major facets of cognitive errors. Specifically, the quadrant ‘T’ is placed appropriately as the first one since this represents the first priority to check against possible fatal disease processes. The quadrants ‘W’ and ‘E’ although distinctly different, are interrelated. This is because reflecting on ‘E’ may stimulate the consideration of other possibilities and, hence, a clinician may need to go back to ‘W’. Finally, the quadrant ‘D’ serves as an internal double check, prompting a clinician to ask: “Is there any other reason I need to slow down?” The quadrant ‘D’ also helps the clinician to acknowledge any possible internal or external pressures that a clinician is going through that may affect the quality of the decision made. However, as the cognitive errors represented in quadrant ‘D’ may not be easily recalled and can be confusing for practicing clinicians as it involves an additional step of running through the 2 ‘E’s, greater amount of time and effort may be needed to explain this quadrant to a novice clinician (Additional file 1).

Second, the TWED checklist should be used only after a working diagnosis has been established. As aptly stated by one of the content experts (JR): “[The TWED checklist] really should be (applied) after some form of an initial diagnostic impression is made intuitively. Otherwise it is unlikely to be an efficient process.” Nonetheless, whilst the domains “**W-E-D**” should be used right before the closing of the decision making process, the domain “**T**” should be used at the very outset of the process of generating working diagnoses.

Third, the TWED checklist should be used on every case until it becomes habitual. It can easily be memorized and used regularly for rapid screening to determine whether a more robust cognitive exercise is needed. Furthermore, the subtlety of cognitive errors is that these errors often take place unconsciously. As a result, clinicians may not be aware that they are at risk of committing cognitive errors and should therefore use the checklist, or its mental equivalent, for every case. In fact, as pointed out by another expert (MG), “The cases where the doctor is most sure of are in fact, the cases where the checklist would be most helpful. This is because, when the clinician is puzzled by a case, the clinician would automatically be applying Type II process (analytical thinking) and would be thinking more broadly.”

Fourth, the TWED checklist does not lead to oversimplification. As it should be used only after the generation of an initial impression, it does not actually interfere with the cognitive process itself. Rather, the TWED checklist reinforces the clinical decisions already made. Furthermore, it is expected that clinicians would not be using the TWED checklist alone. Like most mnemonics, it is more of an adjunct to the normal clinical reasoning process. And even if it does lead to oversimplification, as one expert (RT) puts it: “Having this simple application for a complex task is better than omitting the task entirely”.

Finally, the TWED checklist is useful for novice clinicians. The usefulness of this checklist is agreed upon by all the experts who participated in this evaluation process. Three experts even believe that TWED checklist does have some use also among experienced clinicians because essentially what the TWED checklist strives to achieve is on developing good habits. Nonetheless, novice and experienced clinicians may use the checklist differently. Novice clinicians who regularly use the TWED checklist will generally be able to integrate its contents as part of their clinical habits as they mature to become more experienced clinicians, whereas the experienced physicians will use it as a form of double-check mechanism in regulating their clinical decisions.

## Discussion

In contrast to the previous checklists identified from literature review, the TWED checklist is structured in a mnemonic format, facilitating its portability and potentially its use in the clinical setting. The advantage of a mnemonic format is that it aids prospective memory by transforming the technical terms [23] of common cognitive biases into four memorable questions (represented by the four quadrants) that enhances activation of implementation intention.

The brevity of the TWED checklist is an advantage as it helps the clinician to focus on the most pertinent activating questions only. By focusing on these most pertinent questions, this allows the clinician the flexibility to exercise his or her own judgment and to continue using any other strategies the clinician has already been using to reduce cognitive errors. A checklist that is too long with too many additions may render it redundant and useless [16, 24]. More importantly, the brevity of the TWED checklist makes it “portable”. It can be “carried along” with the clinician during his or her multiple patient encounters. It can be applied quickly and repetitively for every clinical case. These repetitive practices nurture the habit of reducing cognitive errors into an automatized routine and by then, the clinician would probably no longer need to rely on the TWED checklist.

For content validation, although most of the categories of cognitive errors represented in the TWED checklist were rated as “excellent” in terms of their representativeness and relevance, the categories of “cognitive errors due to erroneous estimation or perception of prevalence” and “cognitive errors associated with patient characteristics” were rated slightly lower in terms of their relevance in clinical settings. This could be due to the fact that compared to novice clinicians, more senior clinicians are more familiar with the prevalence of common disease processes in their patient populations and were more objective in their clinical evaluations.

For face validity and applicability, the experts believe that the checklist should be applied for every case and practiced repetitively until the checklist is internalized in memory. The majority of the experts believe that the TWED checklist does not lead to oversimplification. In fact, it is the complexity of medical decision making that all the more enhances the need for a simple and brief mnemonic tool like this.

Ideally, the additional diagnoses generated from the checklist should then be subjected to Bayesian analysis to gauge the probability of each of these diagnoses, as not all of these diagnoses should be given the same weight [25].

Several limitations of the studies deserve mentioning. First, the construct of the four quadrants in the TWED checklist was done based on literature review and discussion with only one expert (PC). Construct validity was not quantitatively determined. The lack of construct validation process as well as the reliance on the opinions of a single expert may have introduced personal biases. Second, content validity of the checklist was determined by senior emergency physicians, and the responses of these clinicians may not truly reflect the sentiments of the junior clinicians. Third, the codings for the email responses were performed by a single researcher only (CH) and again, this could have introduced personal biases.

The next step in the TWED checklist development would be to implement it in clinical settings and to study its effects on the prevention of cognitive errors in simulated followed by actual clinical practice. A practical suggestion is to display the TWED checklist in the patient clerking sheet in order to reinforce its use until it becomes a part of the clinician’s cognitive process.

## Conclusion

The TWED checklist is a brief, portable and focused mnemonic checklist, arranged in order of priority with the aim of activating implementation intentions for checking cognitive errors in clinical settings. While its mnemonic structure eases prospective memory and its brevity allows for portability in quick application in every case, its flexibility allows the clinicians to incorporate its use with other forms of cognitive interventions that clinicians are already using.

## Additional file

10.1186/s13104-016-2249-2 Detailed comments on TWED checklist from content experts.
