# Supplementary material for: A portable mnemonic to facilitate checking for cognitive errors
Source: BMC Res Notes. 2016 Sep 17;9:445. doi: 10.1186/s13104-016-2249-2 (PMC5027116; doi:10.1186/s13104-016-2249-2)
Supplement: Supplementary file 1 — 10.1186/s13104-016-2249-2 Detailed comments on TWED checklist from content experts. [file 13104_2016_2249_MOESM1_ESM.docx]

**SUPPLEMENTAL TABLE**

**Detailed Comments On TWED Checklist From Content Experts**

|  | **MG** | **RT** | **JE** | **MS** | **SM** | **PC** | **JR** |
| --- | --- | --- | --- | --- | --- | --- | --- |
| **Do you think that all the important facets of cognitive de-biasing are properly covered in the TWED checklist?** | “All-important aspects are included although not all-inclusive.” | “Major components are covered in TWED. However, the “D = dispositional factors” may not be clear to a typical doctor, especially as there are two-subparts to this quadrant (the 2Es).” | For ‘T’, it would be helpful to have a checklist of rule-out-worst-scenarios (ROWS) for various common symptoms. One should also distinguish between the ‘W’ and ‘E’.  ‘W’ says, "Do I have an adequate differential diagnoses? What other possibilities should I consider?” It would be helpful to have a checklist of the causes for various symptoms when asking these questions (such as the differential diagnoses checklists that can be downloaded in the article by Ely et al, 2011). ‘E basically says, "Do I have sufficient evidence to rule in or rule out a diagnosis?" But how confident do I need to be? What does the patient expect? What would my peers, in similar situation, do? ‘D’ says, "Do I need to be cautious because of affective issues, like anger, fatigue or distraction?" Human beings are terrible at this because we never know when this is happening to us at the time it happens until it's too late. Something about being fatigued or angry makes us blind to the fact that we are too fatigued or angry to do a good job. I have never seen a good strategy to identify these problems when they are occurring. | “Yes, many are covered. However, it misses errors associated with information that are not consistent with the working diagnoses.” | “I would say they are. Both cognitive as well as affective factors involved in most bias-induced errors are addressed in TWED checklist” |  | It covers most of it. For ‘T’, it is appropriate for this to come as the first priority as it is a serious and fatal process. For ‘W’, this not only helps to generate a broader differential diagnoses, but also helps me assign some level of risk to the situation. If the answer to this ‘W’ question is items that bring a significant threat, it is a further motivation to slow down. If the alternative options are benign, I feel more comfortable to proceed on with speed. For ‘E’, I use another way of phrasing this – what fits and what does not. While helping me to define my confidence level in the diagnosis, it may also stimulate me once again to consider other possibilities and go back to step in ‘W’. For ‘D’, it is a good internal double check. Is there any reason I need to slow down? This can be as simple as acknowledging the internal and/or external pressures or stresses. But I just wonder how sticky this part of the mnemonic will be for practicing clinicians, especially on how you can remind clinicians to run through the 2 ‘E’s. It might be a little confusing. |
| **In your opinion, when should a mnemonic checklist to facilitate metacognition such as the TWED checklist be used? Before formulating your initial diagnosis/treatment? Or after?** | Don’t know which would be better. A good area to research” | “This checklist is most appropriately used after the initial diagnostic impression has been formed. Most doctors will be generating and refining diagnostic hypotheses at the bedside and with each piece of additional information from history, physical examination, laboratory or imaging data, adjustment may be made. Once this has been done, systematically running through this checklist formally makes most sense. Although, in actuality, many of the steps in the TWED checklist would already have been used during the clinical reasoning process, this does not obviate the need to use the checklist (but rather the checklist reinforces it).” | “The best time is after the history, physical examination and initial plan formulation.” | TWED checklist could be used after being sure of the working diagnosis, while talking to the patient, and while reorganizing the data in a written or dictated note. Not sure I could use a TWED in practice before I have a diagnosis in mind | “After the initial diagnosis. In most situations, before the doctor can start asking any question from the checklist, an initial diagnosis will probably already be formed in mind, triggered by the exposure to the case findings. In addition, the quadrants “W” and “E” are in relations to the initial diagnosis already considered for the case.” | “I don’t think the timing is critical. Mnemonics are typically used when you are trying to formulate a good differential diagnosis at the work-up stage or later as a checklist to look back on the thinking you have already done. In the case of TWED checklist, however, the first letter “T” should be used at the very outset, whereas “W-E-D” could all be used before closing the thinking process.” | It really should be after some form of an initial diagnostic impression is made intuitively. Otherwise it is unlikely to be an efficient process. To have metacognition, you have to have cognition to be ‘meta’ about. |
| **How often should TWED checklist be used? For every case seen? Or only selective cases? What types of cases, if selective?** | “Every case. The cases where the doctor is most sure of are the cases where the checklist would be most helpful. On the contrary, when the doctor is puzzled by a case, the doctor would automatically be applying Type II process and would be thinking more broadly.” | “It is best used in every case but after a while, it would become part of the usual operating procedure. Many clinicians would be resistant and think that this would be a waste of time, but I am not sure if we are good enough at determining which case we ought to be worried or not worried about.” | “I think every case where the diagnosis is not completely obvious as a doctor is often not very good at knowing when he/she is right. Confidence in diagnosis has correlation with accuracy but still there are many cases where the doctor is confident but wrong.” | “Not sure. It could be incorporated into routine history taking or the impression and plan part of a consult note” | “It should be for every case. This is because it can hardly be assumed that a doctor can determine which of the cases they encounter would require the checklist. The problem is, firstly, biases take place unconsciously, so doctors would not be aware that they are at risk and should therefore use the checklist. And, secondly, case complexity depends on the individual doctor’s expertise, previous experiences etc. A doctor’s judgment about the case difficulty and their confidence in their initial diagnoses have not been shown to be reliable indicators to help them to recognize when further reflection is needed.” | “I think people should always use it. One of the problems in Emergency Medicine is that we sometimes feel too comfortable with simple things – like constipation. We should make it a routine to always go through TWED so that it becomes a habit.” | “A brief one like this can truly be memorized and used regularly to screen for whether a more robust cognitive exercise is needed to improve the accuracy of the process.” |
| **Cognitive process involved in medical decision is highly complex. Do you think that the use of TWED checklist would lead to oversimplification of these cognitive processes?** | “Yes it oversimplifies things but as long as it helps to reduce diagnostic errors, it should be used.” | “No. TWED checklist simply reinforces the use of cognitive techniques that maybe helpful in improving diagnostic performance. Even if it does, this simple application for a complex task is better than omitting the task entirely.” | “Don't know. Whatever debiasing strategy developed should be tested with real patients in real time.” | “No. I think clinicians have overwhelming practice using their own diagnostic processes. I doubt the TWED will supplant these processes.” | “No. If TWED checklist is employed after a working diagnosis is made, it would act as a double-check against eventual flaws in reasoning. It would not, therefore, be an oversimplification as you are not intending that it replaces reasoning processes.” | “No. I would not expect clinicians to only use TWED checklist – like most mnemonics, it is an adjunct to normal clinical reasoning.” | The value |
| **Do you think that the TWED checklist mainly useful for novice doctors, for more experienced ones, or for both groups?** |  | Both. For novices, its use may introduce them to some good practices (albeit unproven effectiveness) while for more experienced doctors, it can reinforce these techniques, prevent complacency and force a cognitive miser into cognitive action. | I think it is for both groups. Novice clinicians need it because they lack the knowledge and experience. Experienced clinicians need it because they may get in a rut and would need to expand their horizons and broaden the differential diagnosis. | More for novice doctors. Experienced clinicians are probably more difficult to motivate to use a cognitive aid, and make fewer errors leaving less room for potential benefit. | Both groups but experienced clinicians could possibly gain more as they tend to rely more often on Type 1 process, and thus may be more prone to bias than novice doctors who tend to reason in a more analytical way. | - |  |

**Expert Panel:**

JR is a nephrologist in Allegheny General Hospital and the Program Director of Internal Medicine Residency Program at Allegheny General Hospital (AGH). His research interests include teaching doctors how to think and the curriculum in cognitive bias and diagnostic error for residents.

PC is Professor and Director of the Critical Thinking Program at the Faculty of Medicine, Dalhousie University, Canada. His research is mainly concerned with clinical decision-making, specifically the diagnostic process.

RT is a patient safety expert and has been in clinical practice for more than 20 years. He is an internist with the Department of Medicine, Maine Medical Center.

JE is Professor Emeritus in the Department of Family Medicine at the University of Iowa Carver College of Medicine in Iowa City.

MG is Professor Emeritus of Medicine, State University of New York & Founder of Society to Improve Diagnosis in Medicine in US

MS is Assistant Professor at McMaster University and interventional cardiologist at Hamilton Health Science. His research interests include medical education and exploring the teaching and practice of clinical decision-making.

SM is an associate professor at the Institute of Medical Education Research Rotterdam, Erasmus Medical Center and the Department of Psychology, Erasmus University Rotterdam, Netherlands.
